# Ophthalmic immune-related adverse events associated with immune checkpoint inhibitors

**DOI:** 10.3389/fimmu.2023.1130238

**Published:** 2023-03-23

**Authors:** Linyang Gan, Huan Chen, Xiaowei Liu, Li Zhang

**Affiliations:** ^1^ Department of Ophthalmology, Peking Union Medical College Hospital, Chinese Academy of Medical Sciences and Peking Union Medical College, Beijing, China; ^2^ Department of Pulmonary and Critical Care Medicine, Peking Union Medical College Hospital, Chinese Academy of Medical Sciences and Peking Union Medical College, Beijing, China

**Keywords:** immune checkpoint inhibitor, adverse events, incidence, inflammation, ophthalmic

## Abstract

**Purpose:**

To investigate the incidence of immune-related adverse events (irAEs) of immune checkpoint inhibitor (ICI) therapy and to report the clinical features, management, and outcomes of ophthalmic irAEs.

**Methods:**

We retrospectively reviewed the medical records of patients who received ICI therapy from January 2016 to September 2022 at Peking Union Medical College Hospital and analyzed the incidence of systemic and ophthalmic adverse effects of this therapy.

**Results:**

Of 962 patients, 248 (25.8%) experienced irAEs. The first-year incidences of total irAEs and ophthalmic irAEs were 23.5% and 1.1%. The most common ICI received by the patients was pembrolizumab (373; 38.8%). Nearly half of the patients (477; 49.6%) had lung cancer. Combination therapy was associated with an increased incidence of irAEs without statistical significance. Patients with lung cancer presented with an increased incidence of total irAEs (p = 0.003) and ophthalmic irAEs (p = 0.032). Eleven patients had ophthalmic manifestations, including ophthalmoplegia (6/11), conjunctivitis (3/11), reactive cutaneous capillary endothelial proliferation (RCCEP) (1/11), and orbital inflammation (1/11). Eight patients had concomitant extra-ophthalmic irAEs. Furthermore, ICIs were discontinued in nine patients, and most ophthalmic manifestations were well controlled with topical and systemic steroids. Ten patients were treated with intravenous or oral steroids. However, cancer progression occurred in five out of eleven patients after the interruption of ICIs.

**Conclusion:**

IrAEs are correlated with ICI regimens and underlying neoplasia. In our Chinese cohort, patients have a higher risk of ophthalmoplegia than uveitis. Early recognition and multidisciplinary consultation are crucial for optimal treatment of ophthalmic irAEs.

## Introduction

Immune checkpoint inhibitor (ICI) therapy has become the first choice for patients with advanced cancer. The targets of currently approved ICIs include programed cell death-1 protein (PD-1), programed cell death ligand-1 protein (PD-L1), and cytotoxic T-lymphocyte associated antigen 4 (CTLA-4) (1). By blocking these pathways using monoclonal antibodies, cytotoxic T cells can be activated to destroy tumor cells. However, overactivation of T cell function also leads to autoimmune toxicities in other organs, known as immune-related adverse events (irAEs). These adverse events can affect almost all organs, including the eye, orbit, and ocular adnexa.

The severity of irAEs range from mild to severe. Ophthalmic irAEs have a relatively low frequency. Approximately 2.8–7.4% of patients receiving ICI therapy experience ophthalmic irAEs (2–5). Although ophthalmic irAEs are usually low-grade and not life-threatening compared with irAEs of other systems, such as myocarditis, they can sometimes severely impact patients’ visual function and cause deterioration in their quality of life (1, 6). However, many patients do not receive timely and appropriate ophthalmic treatment because of insufficient attention. This is because, despite being reported as serious irAEs, only 64% of patients with ophthalmic manifestations are evaluated by ophthalmologists (5). Although most ophthalmic irAEs are well controlled by local and/or oral glucocorticoids (GCs), early recognition and appropriate therapeutic intervention are essential to prevent permanent vision loss, and avoid discontinuation of life-prolonging ICI therapy.

Most data in the literature were based on the White populations (2, 3, 7).In this study, we aim to report the incidence of irAEs, specifically ophthalmic irAEs, in a large group of Chinese patients receiving ICI therapy. Clinical features, management, and outcomes of ophthalmic irAEs are also described.

## Methods

Data on patients receiving ICI therapy from January 2016 to September 2022 were collected retrospectively. Patients were included if they had received an ICI or concurrent chemotherapy to treat a solid tumor or hematological malignancy at Peking Union Medical College Hospital. The ophthalmic diagnoses were confirmed by two ophthalmologists. Patients diagnosed with irAEs in our institution but who had previous ICI therapy at other hospitals were excluded. Medical records were reviewed in detail for cancer history, type of ICIs, time of irAEs onset and treatments received. Patients were included in the analysis if they were followed-up at least 6 months after the first dose of ICIs or if they developed irAEs.

This study was approved by the Institutional Review Board of Peking Union Medical College Hospital and complied with the Declaration of Helsinki. The study only involved the analysis of existing medical data and records. The information of all included patients was anonymized and unidentifiable. Therefore, the requirement for informed consent was waived.

Statistical analysis was performed using SPSS (version 20; IBM, Armonk, NY, USA). The normality of data distribution was confirmed with the Kolmogorov-Smirnov test. Continuous non-normally distributed data were presented as medians (first quartile, third quartile) and compared by non-parametric test. Categorical variables were analyzed by Fisher’s exact test or the chi-square test. A Kaplan-Meier survival curve was performed to show the survival probability. A p-value <0.05 was considered statistically significant.

## Results

**Figure 1:**
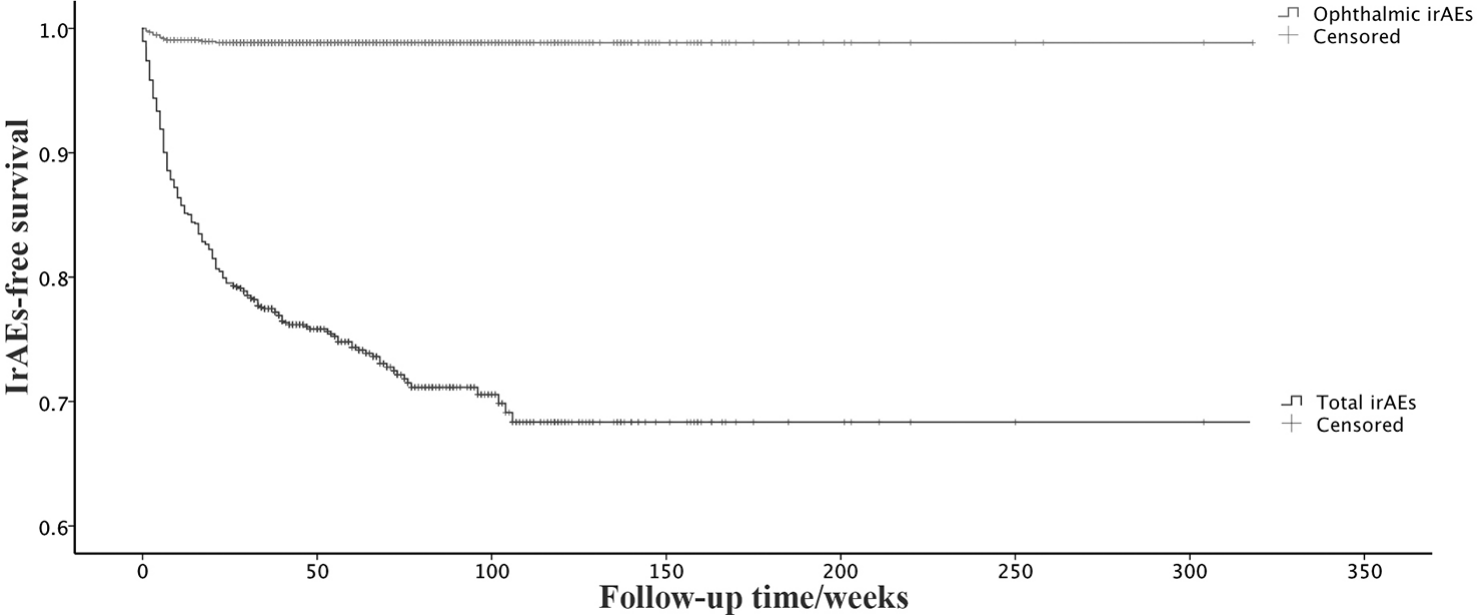
Kaplan–Meier survival curve of total irAEs and ophthalmic irAEs.

Data from 962 patients who had been treated with ICIs were collected (**Table 1**). Of these, 946 patients received PD-1 or/and PD-L1, and 16 patients received CTLA-4 monotherapy or combined with PD-1/PD-L1. The most common ICI used in this study was pembrolizumab (373; 38.8%), followed by tislelizumab (118; 12.3%), sintilimab (98; 10.2%), and camrelizumab (89; 9.3%). About half of the patients (477; 49.6%) had lung cancer. The median follow-up was 53 weeks. We identified 248 (25.8%) patients who developed irAEs, including 11 ophthalmic irAEs. The most common system affected by irAEs was endocrine (78, 31.5%), followed by skin (59, 23.8%) and lung (48, 19.4%). The first-year incidence rates of total irAEs and ophthalmic irAEs were 23.5% and 1.1%, respectively. The onset time of total irAEs ranged from one day to 106 weeks, with a median of 10 weeks (interquartile range 4-22 weeks). Ophthalmic irAEs occurred relatively earlier (median 5 weeks, interquartile range 2-7 weeks) (**Figure 1**).

**Table 1:** Demographic and clinical features of patients receiving ICI therapy.

	All, *N* = 962	Non-irAE, *n* = 714	Total irAE, *n* = 248
**Male, %**	63.4%	60.1%	73.0%
**Age, years**	62 (53, 68)	61 (52, 67)	65 (56, 69)
Type of cancer			
Lung, n (%)	477 (49.6%)	334 (46.8%)	143 (57.7%)
Gastrointestine, n (%)	135 (14.0%)	102 (14.3%)	33 (13.3%)
ENT, n (%)	41 (4.3%)	33 (4.6%)	8 (3.2%)
Urothelial tract, n (%)	56 (5.8%)	43 (6.0%)	13 (5.2%)
Cervix, n (%)	53 (5.5%)	45 (6.5%)	8 (3.2%)
lymphoma, n (%)	33 (3.4%)	29 (4.1%)	4 (1.6%)
Ovary, n (%)	39 (4.1%)	31 (4.3%)	8 (3.2%)
*Other tumors, n (%)	128 (13.3%)	97 (13.6%)	31 (12.5%)
ICI classes			
PD-1/PD-L1, n (%)	946 (98.3%)	704 (98.6%)	242 (97.6%)
CTLA-4, n (%)	2 (0.2%)	2 (0.3%)	0 (0%)
PD-1/PD-L1+CTLA-4, n (%)	14 (1.5%)	8 (1.1%)	6 (2.4%)
ICI drugs			
Pembrolizumab, n (%)	373 (38.8%)	265 (37.1%)	108 (43.5%)
Tislelizumab, n (%)	118 (12.3%)	89 (12.5%)	29 (11.7%)
Sintilimab, n (%)	98 (10.2%)	73 (10.2%)	25 (10.1%)
Camrelizumab, n (%)	89 (9.3%)	64 (9.0%)	25 (10.1%)
Nivolumab, n (%)	59 (6.1%)	47 (6.6%)	12 (4.8%)
Toripalimab, n (%)	46 (4.8%)	41 (5.7%)	5 (2.0%)
Durvalumab, n (%)	40 (4.2%)	29 (4.1%)	11 (4.4%)
Atezolizumab, n (%)	27 (2.8%)	20 (2.8%)	7 (2.8%)
Others, n (%)	112 (11.6%)	86 (12.0%)	26 (10.5%)

ENT—this group includes nasopharyngeal squamous cell carcinoma, Laryngocarcinoma, hypopharyngeal carcinoma. ICIs, immune checkpoint inhibitors; PD-1, programed death-1; PD-L1, programed death-ligand 1.

*Other tumors included 30 cases of choriocarcinoma, 18 hepatobiliary cancer, 17 kidney cancer, 17 endometrial cancer, 9 malignant mesothelioma, 6 thymic carcinoma, 4 pancreatic cancer, 4 glioma, 3 thyroid carcinoma, 2 melanomas, 2 clear cell carcinoma, 1 Merkel cell tumor,1 prostatic cancer, 5 sarcoma, 4 squamous cell carcinoma, 5 metastatic carcinoma of unknown origin.

Combination therapies of CTLA-4 and PD-1/PD-L1 resulted in higher incidences of total irAEs (42.9%) compared with monotherapy (27.6%) and ICIs plus chemotherapy (24.8%). However, no statistically significant differences were detected among these regimens (p = 0.218). Total irAEs were comparable among different ICIs (p = 0.341). Patients with lung cancer seemed to have a higher risk of irAEs. Out of 477 cases of lung cancer, 143 cases of irAEs (30%) were recorded, while of the remaining 485 cases of other cancers, 105 cases of total irAEs (21.6%) were recorded (p = 0.003). A similar difference was also found in ophthalmic irAEs (p = 0.032). Of the 11 recorded ophthalmic irAEs, 9 occurred in patients with lung cancer.

The age, gender, type of cancer, and ICI of 11 patients with ophthalmic irAEs are shown in **Table 2**. Only one patient was female. None had a previous history of ophthalmic inflammatory diseases. Nine patients had advanced lung cancer; the other two patients had colon cancer and esophageal squamous cell carcinoma, respectively. All patients received anti-PD-1 anti-bodies (four camrelizumab, four pembrolizumab, and singular sintilimab, tislelizumab, and nivolumab). The median time from starting ICI therapy to the onset of ophthalmic irAEs was 5 weeks (interquartile range 2-7 weeks). Nine patients had bilateral eye involvement. Eight patients had concomitant irAEs of other organs, mainly myositis and myocarditis.

**Table 2:** Clinical features, treatment, and outcomes of ophthalmic irAEs.

ID	Sex/Age	Cancers	ICIs (cycles)	Onset (weeks)	Ophthalmic irAEs	Initial VA	Systemic irAEs	Treatment	Discontinuation of ICIs	Ophthalmic outcomes	Final VA	Neoplastic outcomes	Follow-up (weeks)
1	M/56	Small cell lung cancer	Camrelizumab (3)	6	Limitation of left abduction	OD 0.6 OS 0.6	None	Oral steroids	Discontinued because of ophthalmic irAEs	Resolved	OD 0.6 OS 0.8	Partial remission	35
2	M/52	Lung adenocarcinoma	Camrelizumab (2)	5	Bilateral ptosis, Limitation of bilateral abduction	OD 0.4 OS 0.4	Myositis, hepatitis	IV steroids, IVIG	Discontinued because of ophthalmic and systemic irAEs	Resolved	OD 0.6 OS 0.6	Stable	54
3	M/70	Small cell lung cancer	Camrelizumab (6)	17	Bilateral blepharitis, conjunctivitis	OD 0.4 OS 0.5	Lichenoid eruptions, oral ulcer	Oral/topical steroids	Discontinued because of systemic irAEs	Resolved, but developed cicatricial ectropion	OD 0.4 OS 0.4	Partial remission	90
4	M/68	Small cell lung cancer	Camrelizumab (2)	5	RCCEP of left eye lid	OD 0.5 OS 0.4	None	Surgical resection	Continued	Resolved	OD 0.5 OS 0.4	Partial remission	42
5	M/62	Lung adenocarcinoma	Pembrolizumab (7)	21	Bilateral periorbital edema, proptosis,	OD 0.6 OS 0.8	None	Oral steroids	Continued	Resolved	OD 0.6 OS 0.8	Progression	43
6	F/63	Small cell lung cancer	Pembrolizumab (1)	2	Bilateral ptosis	OD 0.6 OS 0.7	Myositis, myocarditis, hepatitis	IV steroids, IVIG, MMF	Discontinued because of ophthalmic and systemic irAEs	Partially resolved	OD 0.6 OS 0.7	Stable	35
7	M/78	Lung adenocarcinoma	Pembrolizumab (1)	1	Bilateral conjunctivitis	OD 0.8 OS 0.8	Myocarditis	IV/topical steroids	Discontinued because of systemic irAEs	Resolved	OD 0.8 OS 0.8	Progression	48
8	M/65	Esophageal squamous cell carcinoma	Pembrolizumab (1)	1	Bilateral conjunctivitis	NA	Steven-Johnson syndrome	IV/topical steroids, IVIG	Discontinued because of systemic irAEs	Resolved	NA	Progression	9
9	M/70	Colon cancer	Sintilimab (1)	3	Right ptosis, Limitation of bilateral abduction	OD 0.4 OS 0.25	Myositis	Oral steroids	Discontinued because of ophthalmic and systemic irAEs	Partially resolved	OD 0.4 OS 0.25	Progression	39
10	M/69	Lung Squamous Cell Cancer	Tislelizumab (1)	3	Right ptosis	NA	Myositis, myocarditis	IV steroids, IVIG, MMF, Tocilizumab	Discontinued because of ophthalmic and systemic irAEs	Unresolved	NA	Death	3 days
11	M/65	Lung Squamous Cell Cancer	Nivolumab (2)	7	Limitation of left abduction	OD 0.3 OS 0.5	CN V palsy	Oral steroids	Discontinued because of ophthalmic irAEs	Resolved	OD 0.3 OS 0.5	Progression	9

VA, visual acuity; IV, Intravenous; IVIG, Intravenous immunoglobulin; MMF, Mycophenolate mofetil; CN, cranial nerve; RCCEP, reactive cutaneous capillary endothelial proliferation; NA, not available.

Ophthalmoplegia was the most common ophthalmic irAE in this study (6/11). Ophthalmoplegia occurred a median of 4 weeks after the initiation of ICI treatment. Among these, three patients presented with ptosis, one patient had diplopia, and two suffered from both. Four patients had mucocutaneous involvement. Conjunctivitis was observed in three patients, with one of them having concomitant blepharitis. Patients with conjunctivitis had the shortest onset time of one week. All of these patients were treated with topical steroids for ocular surface inflammation. One patient had reactive cutaneous capillary endothelial proliferation (RCCEP) of the left eyelid after 2 cycles of camrelizumab. One patient in our cohort experienced orbital inflammation. He developed bilateral proptosis and periorbital edema 18 weeks after initiation of pembrolizumab treatment. Orbital MRI showed swelling of the orbital fat and periorbital tissue.

Eight patients had their ICIs discontinued immediately. One patient had camrelizumab discontinued 3 weeks later when his symptoms severely deteriorated after another ICI treatment cycle. The decision of discontinuation was made because of ophthalmic irAEs in two patients, because of extra-ophthalmic irAEs in three patients and because of both in four patients. The other two patients did not stop ICI therapy for their irAEs.

Three patients with ocular surface diseases received topical steroids. Ten patients with ophthalmic irAEs were treated with oral steroids. The initial dosage of oral steroids was 30-50 mg/day. Intravenous methylprednisolone (160-480 mg/day) was used in five patients before oral administration. The median duration of steroids treatment was 4.5 weeks (ranging from two weeks to four months). Four patients with severe extra-ophthalmic irAEs (myositis, myocarditis, and Steven-Johnson syndrome) received intravenous immunoglobulin (IVIG, 15-30g/day X 5 days) concurrently. Mycophenolate mofetil (0.75g bid) was administered to two patients.

At the end of the follow-up, ophthalmic irAEs were well controlled in ten out of eleven patients. Of these, eight patients had complete ophthalmologic remission, and two had partial remission. In terms of cancer control, three patients were in partial remission of neoplasia, one patient had stable disease, five patients’ cancers progressed. One patient died of uncontrolled myocarditis, the outcomes of ophthalmic irAEs and underlying cancer were not available.

## Discussion

ICIs have become a powerful tool in the treatment of advanced malignancy, and their immune-related adverse events are very common (2). Most published research data were generated from the White populations (2, 3, 7). Ethnicity has also been reported as an important factor that influences ophthalmic irAEs (1). The percentage of uveitis-specific ophthalmic irAEs was higher in non-White patients (9.7% in Black patients) compared with 3.5% among White patients [3]. Ophthalmoplegia is more frequently detected in Asians than in Caucasians (1). In this study, we analyzed a cohort of the Chinese population and presented the incidence of total irAEs and ophthalmic irAEs.

Combination therapy with ICIs and chemotherapy or two types of ICIs are more beneficial for prolonging life expectancy in patients with metastatic malignancy, but they also result in a higher incidence of irAEs (8). A recent study reported that combination therapy (ICIs plus chemotherapy or two types of ICIs) caused a three- to four-fold increase in irAE risks (9). Our data showed a higher incidence of irAEs with CTLA4 and PD-1/PD-L1 combination therapy without statistical significance. CTLA4 inhibition results in a high incidence of dose-dependent toxicities, and 38.6-57.9% of patients with melanoma who received ipilimumab developed high-grade toxicities (10). While PD-1/PD-L1 antibodies cause high-grade irAEs in only 10–15% of patients (10). This is consistent with the fact that CTLA-4 blockade activates prime T cells throughout the body, whereas PD-1 and PD-L1 antibodies enhance peripheral antitumor immune cell activity more specifically (2). Combination therapy of ipilimumab has been shown to lead to a twice higher incidence of irAEs than monotherapy (11), which seems to be partially consistent with our data. However, the majority of our cohort received anti-PD-1/PD-L1 agents, only 16 patients had CTLA4 blockade treatment. We might have needed more cases with CTLA4 treatment to reach statistically meaningful conclusions.

Uveitis is considered to be the most common ophthalmic irAE (2, 8, 12, 13). However, we recorded no cases of uveitis in this cohort. To the best of our knowledge, most published cohorts contain large numbers of melanoma patients, ranging from 25.1–67% (14, 15). A registry study in Denmark reported 14 cases of ocular inflammation among 2190 cancer patients, with uveitis in nine out of these 14 cases, occurring among 550 melanoma patients (14). On the other hand, in a case series of ICI-related uveitis, the percentage of melanoma was as high as 76.15–86.7% (2, 6, 7, 16). Uveitis in melanoma patients is thought to be due to the cross reactivity of malignant melanoma cells and normal choroidal melanocytes (8, 16). Similarly, vitiligo is common in melanoma patients treated with ICIs (17), as the skin also contains many melanocytes. Sun et al. proposed that there may be an underlying difference in uveitis risk in patients with melanoma and non-melanoma malignancies (16). The incidence of melanoma is low in Asian populations. Our cohort had only two cases of melanoma. This may partially explain the absence of ICI-related uveitis in this study.

Despite several reports on ophthalmoplegia, it has not been considered a common irAE in previous case series. The most common neuromuscular irAE associated with ICIs is myositis (18, 19). Ocular symptoms, especially ptosis and diplopia, are frequent in ICI-related myositis (19, 20). In a recent study, Lin et al. found that ophthalmoplegia was the leading ophthalmic irAE in advanced lung cancer, accounting for 40.51% (1). The mechanism of neuromuscular irAEs remains unclear. Cancers with a high tumor mutational burden, such as non-small-cell lung cancer, have been associated with a higher risk of irAEs (21). One report documented that nine T-cell antigens shared between non-small cell lung cancer tissue and skin were identified, suggesting overlapping T-cell antigens as a mechanism for irAEs in lung cancer (22). Similarly, we propose that there might be cross antigens between non-small-cell lung cancer tissues and striated muscle.

Symptoms and signs of anterior uveitis can be mild, or even absent. In clinical practice, only patients with new onset eye complaints are likely to receive ophthalmic evaluation. Consequently, the incidence of uveitis might be underestimated. However, the symptoms of ophthalmoplegia can have a serious impact on the daily life of patients, making visiting the hospital timelier. Most cases of ICI-associated ophthalmoplegia in this study had concomitant myositis or myocarditis. Ophthalmologists should be well aware of these life-threatening underlying diseases.

The American Society of Clinical Oncology (ASCO) guidelines recommended that high-grade irAEs generally warrant discontinuation of ICIs and initiation of high-dose steroids (23). Severe and refractory cases may require infliximab or other immunosuppressive therapy. It is crucial that ophthalmologists and oncologists remain vigilant when irAEs are suspected, and carefully weigh the risks and benefits of discontinuing ICI therapy (2). Moreover, the mainstay of controlling irAEs is immunosuppression with steroids, and the long-term effects of high-dose steroids on cancer survivors have not been fully understood (6). We noted that most patients in this study responded well to topical and oral steroids. However, five patients had progressive disease after discontinuation of ICIs and initiation of steroids. Early recognition of ophthalmic irAEs can ensure adequate controls and possible lowering of their effects by prompt topical or oral steroid therapy, so that administration of life-prolonging ICIs can continue (16). In addition, one patient with orbital inflammation had his ICI agent continued, and his symptoms responded well to oral steroids, indicating that it is not necessary to routinely discontinue ICI therapy in patients who develop ICI-related ophthalmic disorders. The rechallenge of ICIs should be considered for patients with progressive neoplasia.

Pembrolizumab, nivolumab, atezolizumab and durvalumab have been authorized by the Food and Drug Administration (FDA) for numerous cancer therapies (24). Tislelizumab, sintilimab, camrelizumab and toripalimab are domestic anti-PD-1 monoclonal antibodies in China. Due to different study populations of clinical trials and a lack of head-to-head trials, comparisons of irAEs could not be made directly between these drugs in most cases (25). In a recent meta-analysis (26), an indirect comparison was conducted to evaluate sintilimab compared with other PD-1/PD-L1 antibodies, including pembrolizumab, atezolizumab, tislelizumab, camrelizumab and nivolumab. The results showed that any grade or grade ≥3 adverse event was comparable between these drugs. Similarly, in our Chinese cohort, total irAEs were comparable among different PD-1/PD-L1 inhibitors (p = 0.341). Our study may offer a choice for comparing the safety profile of these drugs.

Our study has several limitations. These patients were all referred for ophthalmic consultation by oncologists because of ophthalmic symptoms. Mild ophthalmic irAEs might have been neglected and not included in this study. The actual incidence of irAEs could be underestimated because of insufficient attention. In addition, this is a retrospective study in one center with a limited sample size. Only a few cases in our cohort underwent CTLA4 treatment. The exact relationship between different ICI agents and irAEs cannot be determined.

## Conclusion

Combination therapy may increase the risk of irAEs. The spectrum of irAEs can be affected by ethnicity, cancer, or ICI agents. Ophthalmoplegia is the most common ophthalmic irAE in our Chinese cohort. Prompt recognition of ophthalmic irAEs and referral to an ophthalmologist are essential to ensure prompt diagnosis and close monitoring. Multidisciplinary consultation is crucial for optimal treatment and for determining whether to discontinue life-prolonging immunotherapy.

## Data availability statement

The original contributions presented in the study are included in the article/supplementary material. Further inquiries can be directed to the corresponding authors.

## Ethics statement

The studies involving human participants were reviewed and approved by the Institutional Review Board of Peking Union Medical College Hospital. Written informed consent for participation was not required for this study in accordance with the national legislation and the institutional requirements.

## Author contributions

LG, XL and LZ collected the data, LG and HC analysed the data. LG wrote the paper. All authors contributed to the article and approved the submitted version.
